# HPLC–UV assay for the evaluation of inhibitors of plasma amine oxidase using crude bovine plasma

**DOI:** 10.1080/14756366.2018.1524890

**Published:** 2018-11-14

**Authors:** Kira Mergemeier, Florian Galster, Matthias Lehr

**Affiliations:** Institute of Pharmaceutical and Medicinal Chemistry, University of Münster, Münster, Germany

**Keywords:** Plasma amine oxidase, vascular adhesion protein-1, inhibitor screening, bovine plasma, HPLC

## Abstract

Recently, we have described a method for evaluation of plasma amine oxidase (PAO) inhibitors, which monitors the formation of 6-(5-phenyl-2*H*-tetrazol-2-yl)hexanal from the corresponding amine substrate by HPLC with UV-detection using purified bovine PAO. We now investigated, whether crude bovine plasma can be used as enzyme source in this assay instead of the purified enzyme. With the aid of specific inhibitors, it was ensured that there was no detectable activity of other important amine oxidases in the plasma, namely monoamine oxidase (MAO) A and B and diamine oxidase (DAO). For a series of ω-(5-phenyl-2*H*-tetrazol-2-yl)alkan-1-amine substrates similar conversion rates were measured for both the purified PAO and crude plasma. The inhibition values determined for the PAO inhibitor 2-(4-phenylphenyl)acetohydrazide (**16**) under different conditions also corresponded. Additionally, inhibition data of the known PAO inhibitor 2-amino-*N*-(3-phenylbenzyl)acetamide (**17**) and a newly synthesised *meta*-substituted derivative of **16** were determined, which together reflect the two-step inhibition mechanism of these covalent inhibitors.

## Introduction

Plasma amine oxidase (PAO) is a topaquinone- and copper-dependent oxidase, which converts primary amines to aldehydes with the concomitant production of ammonia and hydrogen peroxide^1^. The enzyme, which is also known as copper-containing amine oxidase 3 (AOC3), semicarbazide-sensitive amine oxidase (SSAO) or vascular adhesion protein-1 (VAP-1), is mainly expressed in endothelial cells of blood vessels, smooth muscle cells, and adipocytes. Besides, a soluble form is found in blood plasma, resulting from proteolytic cleavage of membrane-bound vascular PAO^2–4^. The enzyme participates in several physiological and pathophysiological processes^5–7^. In particular, it functions as a vascular adhesion protein that mediates recruitment and extravasation of leukocytes at sites of inflammation^8^^,^^9^. Furthermore, it is involved in glucose transport in adipose cells. Because of the production of reactive aldehydes and hydrogen peroxide, PAO activity is linked to cellular damage in diabetes. Thus, inhibitors of this enzyme could be of therapeutic value in the treatment of inflammatory diseases and diabetic complications^10^^,^^11^.

For the determination of activity of PAO and for screening of inhibitors of the enzyme, a variety of methods have been reported. Mostly benzylamine is used as substrate and the formation of the enzyme product benzaldehyde is measured directly by UV-spectroscopy at 250 nm^12–17^ or by HPLC with UV-detection after derivatisation with dinitrophenylhydrazine^18^. Alternatively, ^14^C-benzaldehyde produced from ^14^C-benzylamine is quantified radiometrically^19^^,^^20^. Furthermore, hydrogen peroxide formed during benzylamine transformation is determined in enzyme-coupled colorimetric^21–26^ or fluorometric assays^27–31^. Besides, a direct fluorimetric method using a (naphthalene-2-yl)methylamine substrate was published^32^. A disadvantage of the methods using benzylamine as substrate is that this substrate only weakly binds to the enzyme. This property could lead to an overestimation of the inhibitory potency of inhibitors when the assay is performed under competitive conditions^33^. We have recently developed an HPLC/UV-method for evaluation of PAO inhibitors, which uses the new tightly binding substrate 6-(5-phenyl-2*H*-tetrazol-2-yl)hexan-1-amine^33^. Although the physiological substrates of PAO are not clearly defined presently, it can be assumed that inhibition data are more meaningful applying tightly instead of weakly binding substrates.

In our published PAO assay, we used the enzyme isolated and purified from bovine plasma. Since the supplier of the enzyme could not deliver the product for some time, we now studied, whether bovine plasma itself can be used for inhibitor screening instead of the purified enzyme. With the modified assay the inhibition properties of two known and one new covalent binding PAO inhibitors were compared applying varying incubation conditions to gain more insights into the factors determining PAO inhibition.

## Materials and methods

### Chemistry

*General*. Column chromatography was performed on silica gel 60, particle size 0.040–0.063 mm, from Macherey & Nagel (Düren, Germany). The melting point of the target compound **18** was determined on a Büchi B-540 apparatus (Essen, Germany) and is uncorrected. The ^1^H-NMR and ^13^C-NMR spectra were recorded on an Agilent DD2 600 spectrometer (600 MHz) (Richardson, TX). The high-resolution mass spectrum (HRMS) was measured on a Bruker micrOTOF-Q II spectrometer (Bremen, Germany) applying atmospheric pressure chemical ionisation (APCI). The purity of **18** was determined by reversed phase HPLC on a Macherey & Nagel Nucleosil 100 RP18 column (3 mm inside diameter × 125 mm, particle size 3 µm) (Düren, Germany) at a flow rate of 0.4 mL/min with a gradient consisting of acetonitrile/aqueous TRIS solution (50 mM, pH 8.5 at 20 °C) (5:95 to 90:10, v/v). The sample was prepared by mixing 20 μL of a 5 mM solution of the compound in DMSO with 180 μL of acetonitrile. An aliquot of 5 μL of this solution was injected into the HPLC-system. The temperature of the column oven was 20 °C. UV-absorbance was measured at 254 nm.

*2-(3-Phenylphenyl)acetohydrazide (****18****).* Hydrazine monohydrate (273 mg, 5.45 mmol, 265 µL) was added to a solution of ethyl 2-(3-phenylphenyl)acetate^34^ (140 mg, 0.58 mmol) in ethanol (7 mL) and the mixture was heated to reflux for 42 h. Further amounts of hydrazine monohydrate were added after 19 h (273 mg, 5.45 mmol, 265 µL) and 27 h (273 mg, 5.45 mmol, 265 µL). After cooling the mixture to ambient temperature, the solvent was removed under reduced pressure. The crude product was treated with a small amount of ice-cold ethanol. The precipitate obtained was filtered off by suction and washed twice with ice-cold ethanol to give **18** as a white solid (39 mg, 29%). C_14_H_14_N_2_O (226.3); mp 126 °C; ^1^H-NMR (600 MHz, DMSO-d_6_): δ (ppm) 3.42 (s, 2H), 4.24 (s, 2H), 7.25 (dt, *J* = 7.7 Hz and 1.4 Hz, 1H), 7.35–7.40 (m, 2H), 7.45–7.49 (m, 2H), 7.51 (ddd, *J* = 7.7 Hz, 1.9 Hz and 1.1 Hz, 1H), 7.55 (t, *J* = 1.8 Hz, 1H), 7.61–7.64 (m, 2H), 9.23 (s, 1H); ^13^C-NMR (151 MHz, DMSO-d_6_): δ (ppm) 40.55, 124.78, 126.66, 127.42, 127.43, 128.03, 128.80, 128.92, 136.93, 140.14, 140.17, 169.51; HRMS (APCI, direct probe) *m/z* [M + H]^+^ calculated: 227,1179, found: 227,1163. Purity (HPLC) 99%.

### Biochemistry

*Reagents.* Heparin-sodium 25,000 I.U./5 mL (Ratiopharm, Ulm, Germany); phosphate buffered saline (PBS) tablets (one tablet dissolved in 200 mL of deionised water yields 0.01 M phosphate buffer, 0.0027 M potassium chloride and 0.137 M sodium chloride, pH 7.4 at 25 °C), dimethyl sulfoxide, benzylamine (**7**), phenamil methanesulfonate salt, clorgiline, selegiline (Sigma-Aldrich, Steinheim, Germany); tris(hydroxymethyl)aminomethane (TRIS) base, benzaldehyde (**14**) (VWR, Darmstadt, Germany); acetonitrile HPLC-grade (Fischer Scientific, Loughborough, UK); bovine PAO purified from bovine plasma (lyophilised powder, activity: 32 Tabor units/mg protein) (Abnova, Taipei, Taiwan delivered *via* Biozol, Eching, Germany); ω-(5-phenyl-2*H*-tetrazol-2-yl)alkanamines **1**–**6**, the corresponding aldehydes **8**–**13** and the PAO inhibitors 2-(4-phenylphenyl)acetohydrazide (**16**) and 2-amino-*N*-(3-phenylbenzyl)acetamide (**17**) were synthesised as published recently;^13^^,^^33^^,^^35^^,^^36^ bovine blood was obtained from Coesfelder Fleischhandelsgesellschaft, Bernd Grewe (Coesfeld, Germany).

*Isolation of bovine plasma.* Immediately after the death of the animal, bovine blood was collected in a 250 mL polypropylene vessel, which contained 5,000 I.U. heparin. The blood was centrifuged in 50 mL Falcon^™^ tubes at 1,500 × *g* for 15 min at room temperature. The plasma supernatant was carefully separated by aspiration and frozen in polypropylene tubes at −20 °C until further use.

*Determination of the conversion rates of different amine substrates by bovine plasma.* The conversion rates of the amine substrates **1**–**7** to the corresponding aldehydes **8**–**14** by bovine plasma was measured as described previously^33^, with the modification that the solution of the isolated PAO in PBS (0.19 mg/mL) (95 µL) was replaced by PBS (89 µL) and bovine plasma (6 µL).

*Determination of the IC_50_-values of the PAO inhibitor 2-(4-phenylphenyl)acetohydrazide (****16****) with bovine plasma using different substrates.* The IC_50_-values were measured as described previously (without pre-incubation),^33^ with the modification that the solution of the isolated PAO in PBS (0.19 mg/mL) (95 µL) was replaced by PBS (89 µL) and bovine plasma (6 µL).

*General procedure for inhibitor screening without pre-incubation of bovine plasma with the inhibitor.* A mixture of a DMSO solution of **4** (10 mM) (2.5 µL), a DMSO solution of the appropriate inhibitor (concentration variable) (2.5 µL) and PBS buffer (pH 7.4) (89 µL) was treated with bovine plasma (6 µL). After incubation at 37 °C for 45 min, the enzyme reaction was terminated by the addition of acetonitrile (100 µL). The sample was cooled in an ice bath for 10 min and centrifuged at 12,000 × *g* and 10 °C for 5 min. An aliquot of the supernatant (150 µL) was diluted with aqueous TRIS buffer (100 mM, pH 8.5 at 20 °C) (150 µL), allowed to stand at room temperature for 30 min, and analysed by HPLC. The HPLC–UV-system consisted of a Thermo Scientific Dionex UltiMate 3000 system including an autosampler and a variable wavelength detector (ThermoFisher Scientific, Germering, Germany). Separation was achieved on a Luna 3 u C8(2) analytical column (3 mm inside diameter× 150 mm, particle size 3 µm) (Phenomenex, Aschaffenburg, Germany) protected with a Phenomenex Phenyl guard column (3 mm inside diameter ×4 mm). The injection volume was 50 µL. Temperature of the autosampler was set to 10 °C, the temperature of the column oven was 20 °C. HPLC analysis was run isocratically using acetonitrile/aqueous TRIS buffer (50 mM, pH 8.5 at 20 °C) (35:65, v/v) as eluent at a flow rate of 0.4 mL/min. The effluents were monitored at 238 nm. Controls with DMSO (2.5 µL) instead of a DMSO solution of the inhibitor (2.5 µL) were prepared in the same manner in parallel (*n* = 3). Furthermore, a blank incubation, in which the enzyme solution was replaced by the buffer used for dissolving the enzyme, was carried out. The relative inhibition values were determined at different inhibitor concentrations and calculated as the ratio of the areas of the 1,3-oxazolidine **15** peaks formed in presence and absence of the test compounds (corrected by the blank value). From these data, the IC_50_-values were calculated *via* Probit-log concentration graphs. The experiments using benzylamine (**7**) as substrate were carried out in the same way with the modifications that aliquots (150 µL) of the supernatants obtained after addition of acetonitrile and centrifugation were diluted with water (150 µL) and acetonitrile/aqueous ammonium acetate solution (10 mM, pH 6) (45:55, v/v) was used as eluent at a detection wavelength of 250 nm.

*General procedure for inhibitor screening with pre-incubation of bovine plasma with the inhibitor.* To a DMSO solution of the appropriate inhibitor (concentration variable) (2.5 µL) was added a dilution of bovine plasma in PBS (95 µL), prepared by mixing 89 volume parts of PBS with 6 volume parts of bovine plasma. The resulting mixture was pre-incubated at 37 °C for 15 min. Then the incubation was started at 37 °C by addition of a solution of the substrate **4** (10 mM) in DMSO (2.5 µL). After 45 min, the enzyme reaction was terminated by the addition of acetonitrile (100 µL). The samples were further treated and analysed as described above in the inhibition experiments done without pre-incubation. Controls (*n* = 3) and a blank were prepared analogously. The experiments using benzylamine (**7**) as substrate were carried out in the same way with the modifications that aliquots (150 µL) of the supernatants obtained after addition of acetonitrile and centrifugation were diluted with water (150 µL), and acetonitrile/aqueous ammonium acetate solution (10 mM, pH 6) (45:55, v/v) was used as eluent at a detection wavelength of 250 nm.

## Results and discussion

According to literature, the high amine oxidase activity in bovine plasma is mainly caused by PAO^37–40^. Other important amine oxidases present in the organism like monoamine oxidase A (MAO A), MAO B, and diamine oxidase (DAO) obviously are not of significant relevance for the cleavage of primary amines in plasma. Therefore, it seemed to be possible to use crude bovine plasma instead of the purified enzyme for the evaluation of PAO inhibitors.

**Figure 1.**
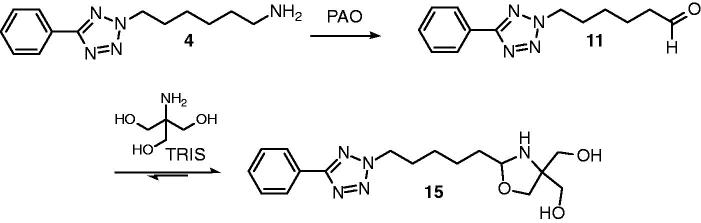
Conversion of 6-(5-phenyl-2*H*-tetrazol-2-yl)hexan-1-amine (**4**) to 6-(5-phenyl-2*H*-tetrazol-2-yl)hexanal (**11**) by PAO and derivatisation of the aliphatic aldehyde product to the 1,3-oxazolidine **15** by TRIS.

During the development and validation of our published method for the detection of PAO inhibitors using purified bovine PAO^33^, we tested a series of phenyltetrazolylalkanamines (Table 1) for their usefulness as substrate. The highest conversion rates were found for the 5-phenyl-2*H*-tetrazol-2-yl-substituted butan-, pentan-, and hexan-1-amines **2**–**4** (Table 1). For the implementation of the assay we chose the hexan-1-amine substrate **4**. The product 6-(5-phenyl-2*H*-tetrazol-2-yl)hexanal (**11**) produced from this compound by PAO was measured by HPLC with UV-detection^33^. Since this aldehyde product only eluted with poor peak shape due to hydrate formation in the aqueous mobile phase, a pre-column derivatisation with tris(hydroxymethyl)aminomethane (TRIS) leading to the formation of a non-hydratable oxazolidine was carried out before analysis (Figure 1).

**Table 1:** Conversion rates of different amines by purified bovine PAO and by bovine plasma.
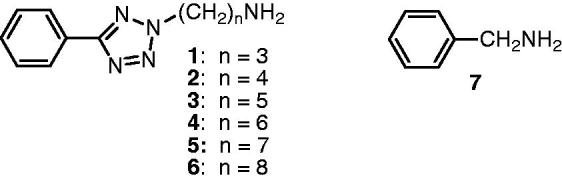

Substrate	Conversion of substrate [%]^a^	
	Purified PAO^b^	Bovine plasma
3-(5-Phenyl-2*H*-tetrazol-2-yl)propan-1-amine (**1**)	12 ± 0.8	5 ± 0.8
4-(5-Phenyl-2*H*-tetrazol-2-yl)butan-1-amine (**2**)	55 ± 2.6	66 ± 5.6
5-(5-Phenyl-2*H*-tetrazol-2-yl)pentan-1-amine (**3**)	45 ± 1.3	51 ± 4.3
6-(5-Phenyl-2*H*-tetrazol-2-yl)hexan-1-amine (**4**)	57 ± 1.4	51 ± 1.5
7-(5-Phenyl-2*H*-tetrazol-2-yl)heptan-1-amine (**5**)	30 ± 1.1	23 ± 2.1
8-(5-Phenyl-2*H*-tetrazol-2-yl)octan-1-amine (**6**)	25 ± 1.2	26 ± 1.8
Benzylamine (**7**)	20 ± 2.1	30 ± 2.0

a The substrate (250 µM) was incubated with the appropriate enzyme (18 µg purified PAO or 6 µL of bovine plasma) in a final volume of 100 µL. The amount of substrate converted was determined by HPLC with UV detection using solutions of the corresponding enzyme products **8**–**14** as previously described;^33^ values are means ± standard deviations, *n* = 3.

b Values already published^33^^,^^35^.

**Figure 2.**
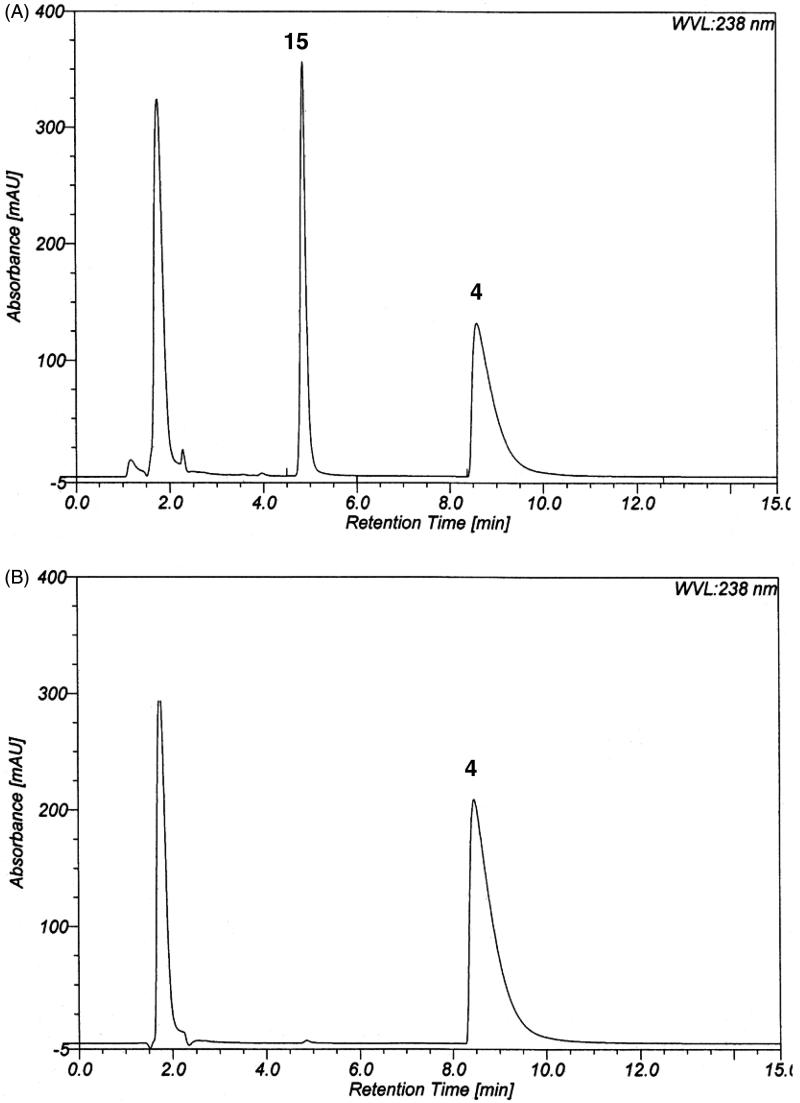
(A) Reversed phase chromatogram of a solution of the substrate 6-(5-phenyl-2*H*-tetrazol-2-yl)hexan-1-amine (**4**) (250 µM) treated with bovine plasma (6 µL per 100 µL sample volume). After incubation at 37 °C for 45 min, the enzyme reaction was terminated by addition of acetonitrile. The sample was centrifuged and diluted with an equal amount of aqueous TRIS buffer (100 mM, pH 8.5 at 20 °C). Chromatography was performed on a Luna 3 μm C8(2) analytical column with acetonitrile/aqueous TRIS buffer (50 mM, pH 8.5 at 20 °C) (35:65, v/v) at a flow rate of 0.4 ml/min using a detection wavelength of 238 nm. (B) Reference chromatogram obtained under the same conditions in absence of bovine plasma (blank). **4**: Amine substrate; **15**: TRIS adduct of aldehyde **11** liberated by the enzyme.

In this work, we first evaluated, whether it is possible to achieve similar conversion rates for the substrate **4** as obtained in the isolated enzyme assay using reasonable amounts of bovine plasma instead of the purified PAO. It was found that 6 µL bovine plasma per 100 µL sample volume produced about the same amount of aldehyde **11** as the applied quantity of purified PAO (18 µg in 100 µL sample volume). Figure 2 shows a chromatogram of the TRIS adduct **15** of the enzyme product **11** released by bovine plasma from the hexan-1-amine substrate **4**. No significant differences to corresponding chromatograms obtained with the purified PAO at the same detection wavelength (238 nm) could be observed. In particular, interfering peaks resulting from the plasma were not visible at the retention time of the 1,3-oxazolidine.

Next, we tested the conversion rates of the related amines **1**–**3** and **5**–**7** by bovine plasma. As shown in Table 1, comparable results were obtained with the purified enzyme and the crude plasma. In both cases, the butane-, pentane-, and hexane-1-amines **2**–**4** were the best substrates with a conversion rate of about 50%. The longer-chain heptane- and octane-1-amines **5** and **6** were transformed only half as efficiently. Their conversion rates corresponded to that of benzylamine (**7**), which is often used as substrate in PAO assays. The short-chain propan-1-amine **1** was reacted by both purified PAO and bovine plasma even less (about 5–10% of the maximum possible amount) under the conditions applied.

To evaluate the comparability of inhibition data obtained with the purified enzyme and the crude plasma, we measured the inhibition values of the known PAO inhibitor 2-(4-phenylphenyl)acetohydrazide (**16**) (for formula see Table 3) using the different amine substrates **1**–**7**. In these experiments, the enzyme was added to a solution containing both substrate and inhibitor. Thus, no pre-incubation of enzyme with the inhibitor took place. As shown in Table 2, all IC_50_-values obtained with the plasma were about twofold higher than the IC_50_-values determined with the purified enzyme. This may be due to the binding of the inhibitor to plasma proteins leading to a somewhat reduced effective concentration. In addition, consistent differences in the apparent inhibitory potency of the inhibitor **16** in dependence of the substrate applied could be registered for both enzyme preparations. The reason for these fluctuating values lies in strongly different binding affinities of the substrates to the enzyme as discussed recently^33^. The parallelism of the inhibition values of **16** on using the purified enzyme and the crude plasma is another proof of that PAO is the enzyme in bovine plasma actually responsible for the cleavage of the used substrates.

**Table 2.** Inhibitory potency of 2-(4-phenylphenyl)acetohydrazide (**16**) against purified bovine PAO and PAO in bovine plasma

Substrate	IC_50_ (µM)^a^	
	Purified PAO	Bovine plasma
3-(5-Phenyl-2*H*-tetrazol-2-yl)propan-1-amine (**1**)	53 ± 3	n.d.^c^
4-(5-Phenyl-2*H*-tetrazol-2-yl)butan-1-amine (**2**)	2.5 ± 0.30^b^	5.3 ± 0.49
5-(5-Phenyl-2*H*-tetrazol-2-yl)pentan-1-amine (**3**)	5.4 ± 0.09^b^	9.7 ± 0.78
6-(5-Phenyl-2*H*-tetrazol-2-yl)hexan-1-amine (**4**)	11 ± 1.2^b^	17 ± 0.80
7-(5-Phenyl-2*H*-tetrazol-2-yl)heptan-1-amine (**5**)	n.d.^d^	n.d.^d^
8-(5-Phenyl-2*H*-tetrazol-2-yl)octan-1-amine (**6**)	11 ± 0.85^b^	16 ± 3.4
Benzylamine (**7**)	0.12 ± 0.03^b^	0.29 ± 0.07

a Inhibition values obtained without pre-incubation of enzyme and inhibitor; means ± standard deviation (*n* = 3).

b Values already published^33^.

c Not determined due to the low conversion rate of **1** in bovine plasma (see Table 1).

d Not determined because the TRIS adduct of the produced aldehyde co-eluted with the inhibitor.

**Table 3.** Inhibitory potency of 2-(4-phenylphenyl)acetohydrazide (**16**), 2-amino-*N*-(3-phenylbenzyl)acetamide (**17**) and 2–(3-phenylphenyl)acetohydrazide (**18**) against PAO in bovine plasma in dependence of the substrate and the assay conditions.
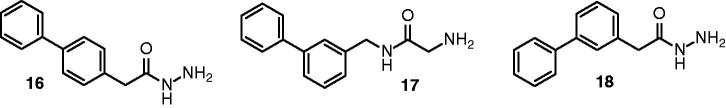

	Inhibitor IC_50_ (µM)^a^		
Conditions and Substrate	**16**	**17**	**18**
Measurement without pre-incubation of enzyme and inhibitor:			
Hexan-1-amine **4**	17 ± 1	0.86 ± 0.04	20 ± 1
Benzylamine	0.29 ± 0.07	0.087 ± 0.003	0.62 ± 0.05
Measurement with pre-incubation of enzyme and inhibitor:			
Hexan-1-amine **4**	0.23 ± 0.04	0.29 ± 0.03	0.38 ± 0.02
Benzylamine	0.13 ± 0.03	0.15 ± 0.01	0.24 ± 0.03

a Values are means ± standard deviations, *n* = 3.

2-(4-Phenylphenyl)acetohydrazide (**16**) is known to inhibit PAO by forming covalent bonds with the cofactor topaquinone. Due to this kind of action, we have studied the inhibition of the purified PAO after pre-incubation of the enzyme with the inhibitor. This procedure provides the inhibitor more time for a covalent modification of the enzyme and its cofactor, respectively, which can lead to a higher inhibitory potency. Applying a pre-incubation time of 15 min, actually the IC_50_-values of **16** against purified PAO using **4** as substrate declined significantly^35^. Furthermore, the pronounced deviation of the inhibition values using **4** and benzylamine, respectively, disappeared. The same effects were observed, when bovine plasma was used (Table 3). Thus, after pre-incubation, the IC_50_-values for **16** were 0.29 µM (substrate **4**) and 0.11 µM (substrate benzylamine) in case of the purified enzyme^35^, and 0.23 and 0.13 µM in case of the analogous experiments with plasma. These data again prove the comparability of the results of the inhibition experiments obtained with the two enzyme sources.

Recently, it was reported that 2-amino-*N*-(3-phenylbenzyl)acetamide (**17**) (Table 3) is a potent covalent inhibitor of PAO^36^. Since we were interested in the inhibitory potency of this compound in comparison to the hydrazide **16**, we re-synthesised **17** and evaluated its activity against PAO in bovine plasma. Performing the measurement without pre-incubation of inhibitor and enzyme and using the phenyltetrazolylhexan-1-amine **4** as substrate, the aminoacetamide **17** was about 20fold more active than the hydrazide **16** (IC_50_-values 0.86 µM vs. 17 µM). With the substrate benzylamine this alteration levelled (IC_50_-values 0.087 µM vs. 0.29 µM). Interestingly, when the inhibitors were pre-incubated with the enzyme no significant differences in the potency of the inhibitors could be seen anymore, whichever of both substrates were used. These results can be explained as follows. The hydrazide **16** and the aminoacetamide **17** are selective irreversible inhibitors reacting with the co-substrate topaquinone *via* their terminal NH_2_-group. Such kind of inhibitors must operate through a two-step mechanism^41^. First, they bind reversibly to the enzyme forming a non-covalent enzyme-inhibitor complex, which can undergo covalent bond formation in a subsequent second step. Without pre-incubation of inhibitor and enzyme, the inhibitors must first compete with the substrate for a reversible binding to the enzyme. When inhibitor and enzyme are pre-incubated, differences in the reversible binding activity no longer play a pronounced role. More important for the observed potency is the reactivity of the nucleophilic group. Obviously, the inhibitor **17** forms much tighter reversible bonds with the enzyme than the inhibitor **16** and the substrate hexan-1-amine **4** much tighter ones than the substrate benzylamine resulting in the strongly varying IC_50_-values observed in the experiments without pre-incubation. The inhibition data of **16** and **17** in the pre-incubation experiments indicate that the reactive groups of these compounds possess about the same reactivity leading to similar IC_50_-values.

Next, we synthesised and tested the hydrazide **18**, in which the reactive functional residue and the terminal phenyl moiety were arranged in *meta*-position like in case of the amide **17**. Compound **18** showed slightly higher IC_50_-values than its *para*-substituted derivative **16** under all test conditions applied (Table 3).

Finally, we wanted to confirm that the amine oxidases MAO A, MAO B, and DAO are not involved in substrate conversion by bovine plasma using specific inhibitorsof these enzymes. Under pre-incubation conditions, the MAO A inhibitor clorgiline and MAO B inhibitor selegiline reduced the activity of MAO A and B, respectively,^35^ by more than 95% at a concentration of 0.1 µM. The DAO inhibitor phenamil produced 43% inhibition of DAO^35^ at 10 µM. In contrast, neither of these compounds blocked the substrate transformation in the assays with purified PAO as well as with bovine plasma at 10 µM under the same conditions, which again proves the central role of PAO for the degradation of primary amines in bovine plasma.

In conclusion, we have shown that crude bovine plasma can be used for PAO inhibitor screening instead of the purified enzyme. Furthermore, it became evident that the inhibition data (IC_50_-values) of the tested covalent inhibitors **16**–**18** strongly depend on the kind of substrate and the incubation conditions used.
